# Efficient delivery of a large-size Cas9-EGFP vector in porcine fetal fibroblasts using a Lonza 4D-Nucleofector system

**DOI:** 10.1186/s12896-023-00799-1

**Published:** 2023-08-16

**Authors:** Weiwei Liu, Xiaoguo Wang, Ruirong Liu, Yaya Liao, Zhiwei Peng, Haoyun Jiang, Qiqi Jing, Yuyun Xing

**Affiliations:** State Key Laboratory of Pig Genetic Improvement and Production Technology, Jiangxi Agricultural University, Nanchang, 330045 China

**Keywords:** Porcine fetal fibroblasts (PFFs), Electroporation, 4D-Nucleofector™, Dual-electroporation program

## Abstract

**Background:**

Porcine fetal fibroblasts (PFFs) are important donor cells for generating genetically modified pigs, but the transfection efficiencies of PFFs are often unsatisfactory especially when large-size vectors are to be delivered. In this study, we aimed to optimize the transfection conditions for delivery of a large-size vector in PFFs using Lonza 4D-Nucleofector™ vessels and strips.

**Methods:**

We firstly delivered a 13 kb Cas9-EGFP and a 3.5 kb pMAX-GFP vector into PFFs via 7 programs recommended by the Lonza basic protocol. We then tested 6 customized dual-electroporation programs for delivering the 13 kb plasmid into PFFs. In addition, we screened potential alternative electroporation buffers to the Nucleofector™ P3 solution. Finally, three CRISPR/Cas9-sgRNAs targeting *Rosa26*, *H11*, and *Cep112* loci were delivered into PFFs with different single and dual-electroporation programs.

**Results:**

Notably lower transfection efficiencies were observed when delivering the 13 kb vector than delivering the 3.5 kb vector in PFFs via the single-electroporation programs. The customized dual-electroporation program FF-113 + CA-137 exhibited higher transfection efficiencies than any of the single-electroporation programs using vessels (98.1%) or strips (89.1%) with acceptable survival rates for the 13 kb vector. Entranster-E buffer generated similar transfection efficiencies and 24-hour survival rates to those from the P3 solution, thus can be used as an alternative electroporation buffer. In the genome-editing experiments, the FF-113 + CA-137 and CA-137 + CA-137 programs showed significantly superior (*P* < 0.01) efficiencies to ones from the single-electroporation programs in vessels and strips. Entranster-E buffer produced higher indel efficiencies than the P3 buffer.

**Conclusions:**

We markedly increased the delivery efficiencies for a large vector via customized dual-electroporation programs using Lonza 4D-Nucleofector™ system, and Entranster-E buffer can be used as an alternative electroporation buffer to Nucleofector™ P3 buffer.

**Supplementary Information:**

The online version contains supplementary material available at 10.1186/s12896-023-00799-1.

## Background

Pigs not only provide one of the most important meat sources globally, they are also among the most useful model animals for biomedical research [1, 2]. With the rapid development of CRISPR/Cas9 technology in recent years, a large number of genetically modified pigs have been produced, most of which being created via somatic cell nuclear transfer (SCNT), with the aim of improving economic traits or creating disease models [3, 4]. In porcine SCNT experiments, genetically modified fibroblasts, such as fetal fibroblasts (PFFs) and ear fibroblasts (PEFs), were extensively utilized as nuclear donor cells [3, 5]. However, transfection of porcine fibroblasts has been a challenge, as porcine fibroblasts were usually considered as hard-to-transfect cell lines [6, 7]. Liposome-mediated transfection usually yields poor transfection efficiencies in porcine fibroblasts [8, 9]; while even though some non-liposome reagents (such as Fugene 6, Fugene HD) have shown better performance[10], the efficiency is still unsatisfactory. Viral transduction has the capability of transducing non-dividing cells and is often with acceptable efficiency, but its use is limited by high cytotoxicity, cargo size limitation, and risk of uncontrolled genome integration [11]. Electroporation is one of the most widely used transfection methods for various mammalian cells [12]. This electro-physical approach can rapidly and efficiently transfect large amounts of cells, and a number of commercial electroporation devices have been developed, such as Neon transfection system, Gene Pulser Xcell Electroporation System, BTX Apparatus, and Lonza’s Nucleofectors. These systems often include versatile strength of electroporation and media used in the process in order to meet the need for transfection of vastly varied cell types.

In recent years PFF cell transfection has become a burden in most of our studies and no method seemed satisfactory. Up to now, a few studies are available on the application of Lonza Nucleofector in porcine cells [13–17], however, no specific optimization of electroporation conditions was mentioned in these studies. In the present study, we attempted to optimize the electroporation conditions for delivering a large-size plasmid in PFF cells using a Lonza 4D-Nucleofector™ system.

## Results

### Transfection efficiencies and cell survival rates using Nucleocuvette™ vessels

**Fig. 1:**
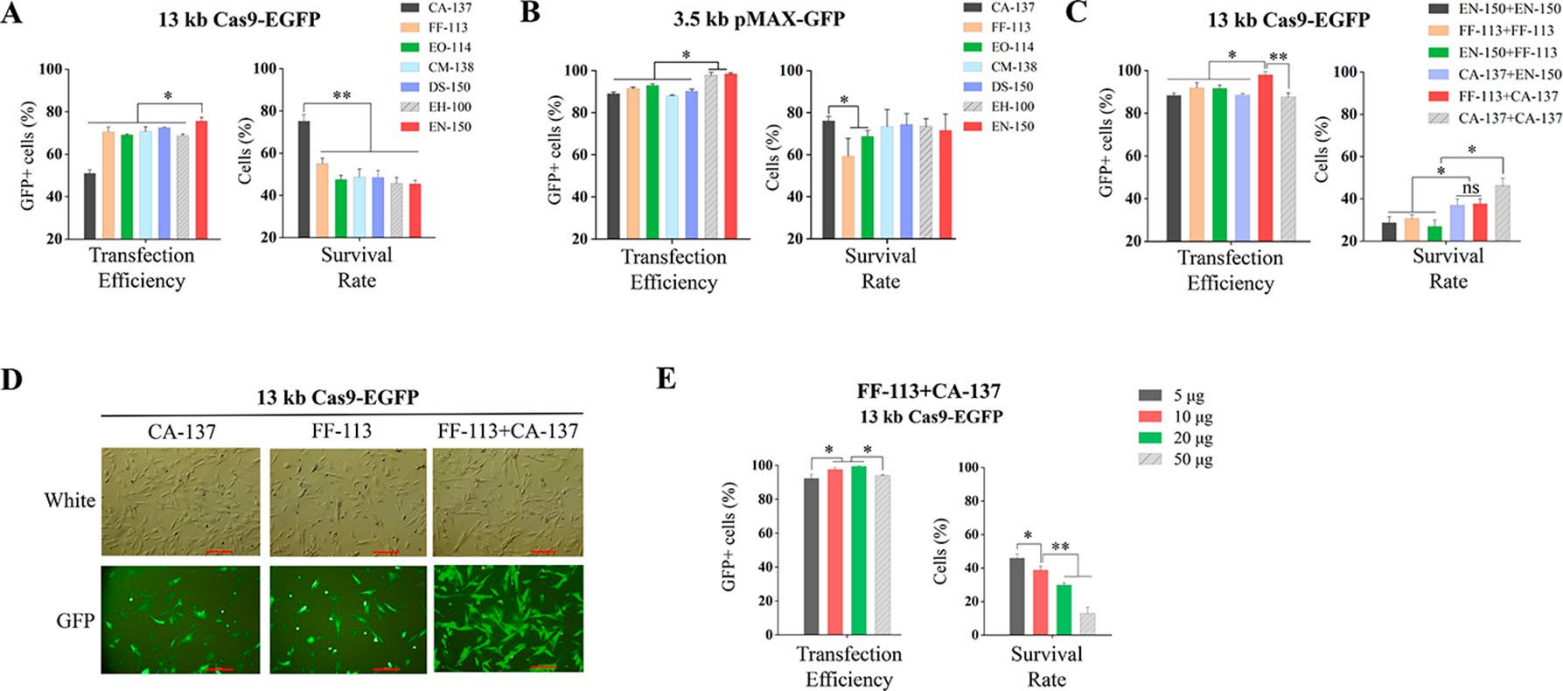
**Electroporation of PFFs using Nucleocuvette™ vessels**. The transfection efficiencies and cell survival rates after delivery of 10 µg Cas9-EGFP (**A**) and equimolar amount of pMAX-GFP (**B**) plasmid via the built-in programs of Lonza basic protocol. (**C**) Transfection efficiencies and cell survival rates after delivery of Cas9-EGFP plasmid via different dual-electroporation programs. (**D**) Representative microscopic photographs 24 h post electroporation. Bars = 200 μm. (**E**) Effect of plasmid dosage on transfection efficiency and cell survival rate. *P < 0.05, **P < 0.01

To test the transfection efficiency of the 4D-Nucleofector™ X unit in PFFs, we firstly delivered 10 µg Cas9-EGFP (13 kb) or equimolar amount of pMAX-GFP (3.5 kb; 2.65 µg) per electroporation reaction using the vessels with the 7 programs recommended by the Lonza Basic Protocol. The results showed that 51.0-75.7% transfection efficiencies were obtained with these seven programs for the Cas9-EGFP plasmid, and survival rates of 45.4–75.1% were observed. The CA-137 program had the lowest transfection efficiency but the highest survival rate, and the EN-150 program showed the opposite results for Cas9-EGFP plasmid (Fig. 1A). In contrast, 88.1–97.9% of transfection efficiencies and 59.4–76.2% of survival rates were observed with these seven programs for the pMAX-GFP (Fig. 1B). Next, EN-150, FF-113, and CA-137 were selected to composite 6 dual-electroporation (consecutive electroporation) programs in an effort to improve the transfection efficiency of the Cas9-EGFP plasmid. With these dual-electroporation programs, significantly higher transfection efficiencies were achieved (87.7–98.1%) at the cost of lower survival rates (26.9–47.0%) (Fig. 1C). The FF-113 + CA-137 program had a transfection efficiency of 98.1% with a survival rate of 37.7%, and the CA-137 + CA-137 program had a transfection efficiency of 87.7% with a survival rate of 47.0% (Fig. 1C). The microscopic images also showed that transfection efficiency was significantly increased via customized FF-113 + CA-137 program compared to CA-137 and FF-113 programs (Fig. 1D). Then the FF-113 + CA-137 program was used to assess the influence of the amount of plasmid on transfection efficiencies. The results showed that 10 and 20 µg of Cas9-EGFP plasmid yielded significantly higher (*P* < 0.05) transfection efficiencies than 5 and 50 µg plasmid. The survival rate significantly decreased following the increase of plasmid doses (Fig. 1E).

### Transfection efficiencies and cell survival rates using Nucleocuvette™ strips

**Fig. 2:**
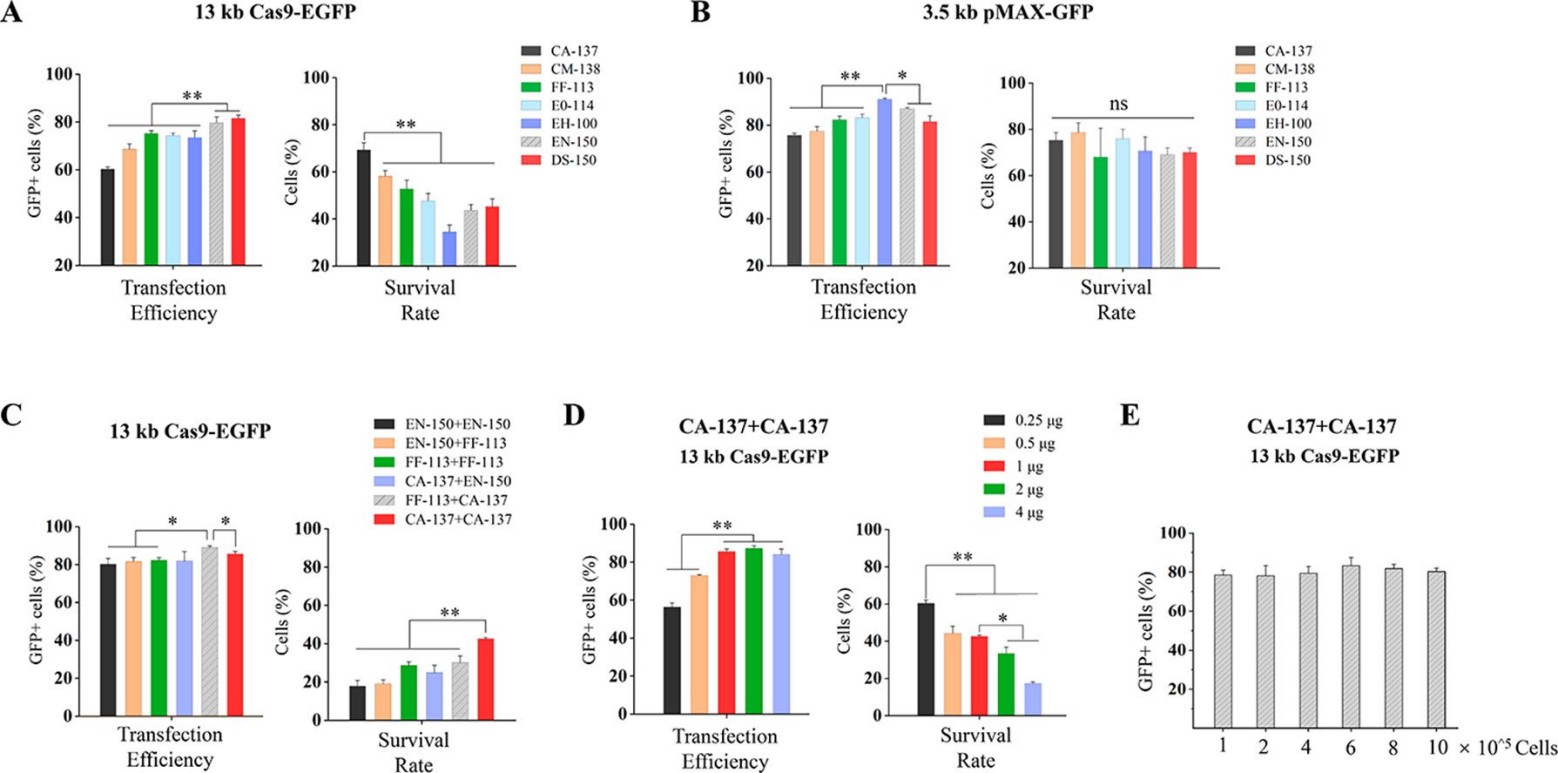
**Electroporation of PFFs using Nucleocuvette™ strips**. The transfection efficiencies and cell survival rates after delivery of 1 µg Cas9-EGFP (**A**) and equimolar amount of pMAX-GFP (**B**) plasmid via the built-in programs of Lonza basic protocol. (**C**) Transfection efficiencies and cell survival rates after delivery of Cas9-EGFP plasmid via different dual-electroporation programs. (**D**) Effect of plasmid dosage on transfection efficiency and cell survival rate. (**E**) Transfection efficiencies after delivery of 1 µg Cas9-EGFP to different amounts of cells. *: *P* < 0.05, **: *P* < 0.01, ns: *P* ≥ 0.05

The seven programs recommended by the Basic Protocol were used to deliver 1 µg of Cas9-EGFP or equimolar amount of pMAX-GFP plasmid (0.265 µg) DNA into PFFs in strips. For the Cas9-EGFP vector, these programs yielded 60.3–81.6% of transfection efficiencies and 34.6–69.3% of survival rates (Fig. 2A), in which the CA-137 program had the lowest transfection efficiency and the highest survival rate. The DS-150 and EN-150 programs showed significantly higher (*P* < 0.05) transfection efficiencies than the other 5 programs, with intermediate levels of survival rates (Fig. 2A). For the pMAX-GFP plasmid, 75.7–91.0% of transfection efficiencies and 68.0-78.7% of survive rates were observed for these seven programs (Fig. 2B). Six customized dual-electroporation programs based on EN-150, FF-113, and CA-137 were executed to determine their transfection efficiencies and survival rates in Nucleocuvette™ strips for the Cas9-EGFP plasmid. The results showed that these programs yielded 80.3–89.1% of transfection efficiencies and 17.8–43.1% of survival rates. The FF-113 + CA-137 program had significantly higher (*P* < 0.05) transfection efficiencies than all the other programs, whereas the CA-137 + CA-137 program had a significantly higher (*P* < 0.05) survival rate than all the other programs (Fig. 2C). To examine the effect of the plasmid amount on transfection, the CA-137 + CA-137 program was used to deliver different doses of Cas9-EGFP plasmid to PFFs in strips. The result showed that the transfection efficiencies using 0.25 and 0.5 µg of plasmid were significantly lower than those using 1, 2, or 4 µg of plasmid (Fig. 2D); cell survival rates decreased along with the increase of the amount of the plasmid. To examine the effect of cell counts on transfection, the CA-137 + CA-137 program was conducted to deliver 1 µg of Cas9-EGFP to different numbers of PFFs (1 × 10^5^-1 × 10^6^), and no significant difference (*P* > 0.05) was found among different groups (Fig. 2E).

### Screening for an alternative electroporation buffer to P3 buffer

**Fig. 3:**
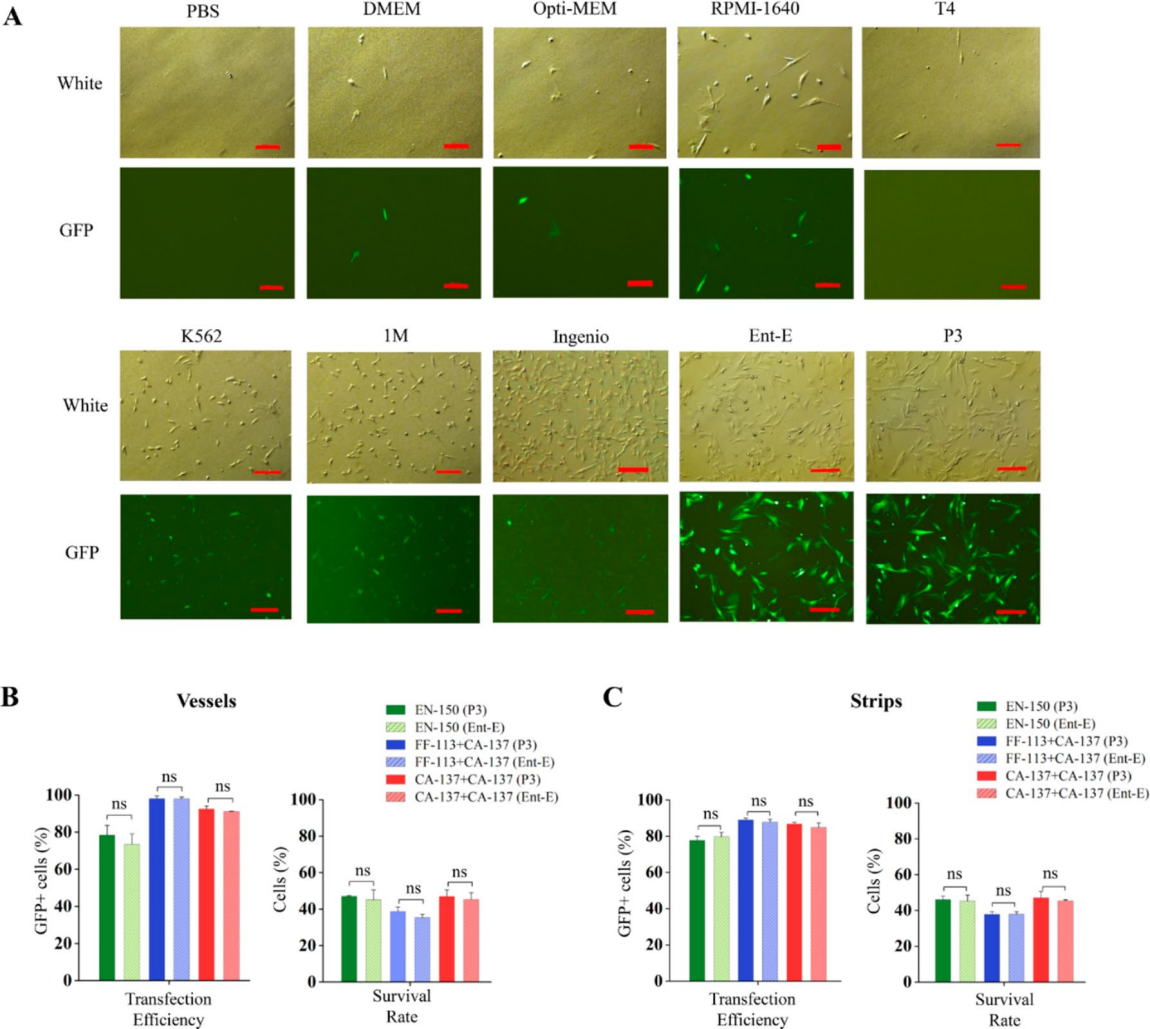
**Screening for an alternative electroporation buffer.** (**A**) Microscopic photographs of electroporation of 13 kb Cas9-EGFP plasmid into PFFs with different buffers. T4: Cytoporation Medium T4; Ingenio: Ingenio® Electroporation Solution; Ent-E: Entranster-E; P3: Lonza 4D-Nucleofector™ X kit P3 buffer. Bar = 200 μm. The cells were electroporated with FF-113 + CA-137 dual-program and the photos were captured 24 h post-electroporation. (**B**) Comparison of transfection effects by delivering the 13 kb Cas9-EGFP plasmid using Entranster-E and P3 buffers in vessels. (**C**) Comparison of transfection effects by delivering the 13 kb Cas9-EGFP plasmid using Entranster-E and P3 buffers in strips. ns: *P* ≥ 0.05

The vessels and strips in the Lonza 4D-Nucleofector™ kit are reusable but the buffer P3 is limited, thus it would be of great help to find an alternate transfection buffer. Inspired by a study[18] that opti-MEM can be used as effective electroporation buffer in the BTX system, we intended to seek out effective and cost-friendly electroporation buffer for the 4D-Nucleofector system. In this study, 4 cell culture media (PBS, DMEM, opti-MEM and RIPA-1640), 2 home-made buffers (K562 and 1 M) used for Lonza 4D-Nucleofector™ in a study [19], 3 commercially available electroporation buffers including Entranster-E that was shown effective for a BTX system in our previous study[20], as well as Cytoporation Medium T4 and Ingenio® Electroporation Solution, were trialed as potential substitute buffers for P3 buffer from the 4D-Nucleofector™ X kit. We firstly compared transfection using these buffers in vessels with the FF-113 + CA-137 program for the13 kb Cas9-EGFP and the EN-150 program for the 3.5 kb pMAX-EGFP. For the 13 kb plasmid, both Entranster-E and P3 buffers resulted in excellent transfection efficiencies as well as survival rates; K562, 1 M and Ingenio® Electroporation Solution achieved low transfection efficiencies and the other buffers resulted in extremely low survival rates (Fig. 3A). For the 3.5 kb plasmid, the Entranster-E and P3 buffers also achieved supreme transfection efficiencies and survival rates, and K562 and RPMI-1640 achieved good transfection efficiencies and survival rates; the rest buffers achieved much lower transfection efficiencies or lower survival rates (Figure S1A). To investigate whether Entranster-E buffer behave similarly in vessels and strips, we further compared the transfection effects by delivering the 13 kb and 3.5 kb plasmids in Entranster-E and P3 buffers in vessels and strips with single and dual-electroporation programs. Entranster-E showed comparable transfection efficiencies and survival rates in all these tests to buffer P3 (Fig. 3B, C; Figure S1 B, C).

### Genome editing efficiencies by delivery of CRISPR/Cas9-sgRNAs using vessels and strips

**Fig. 4:**
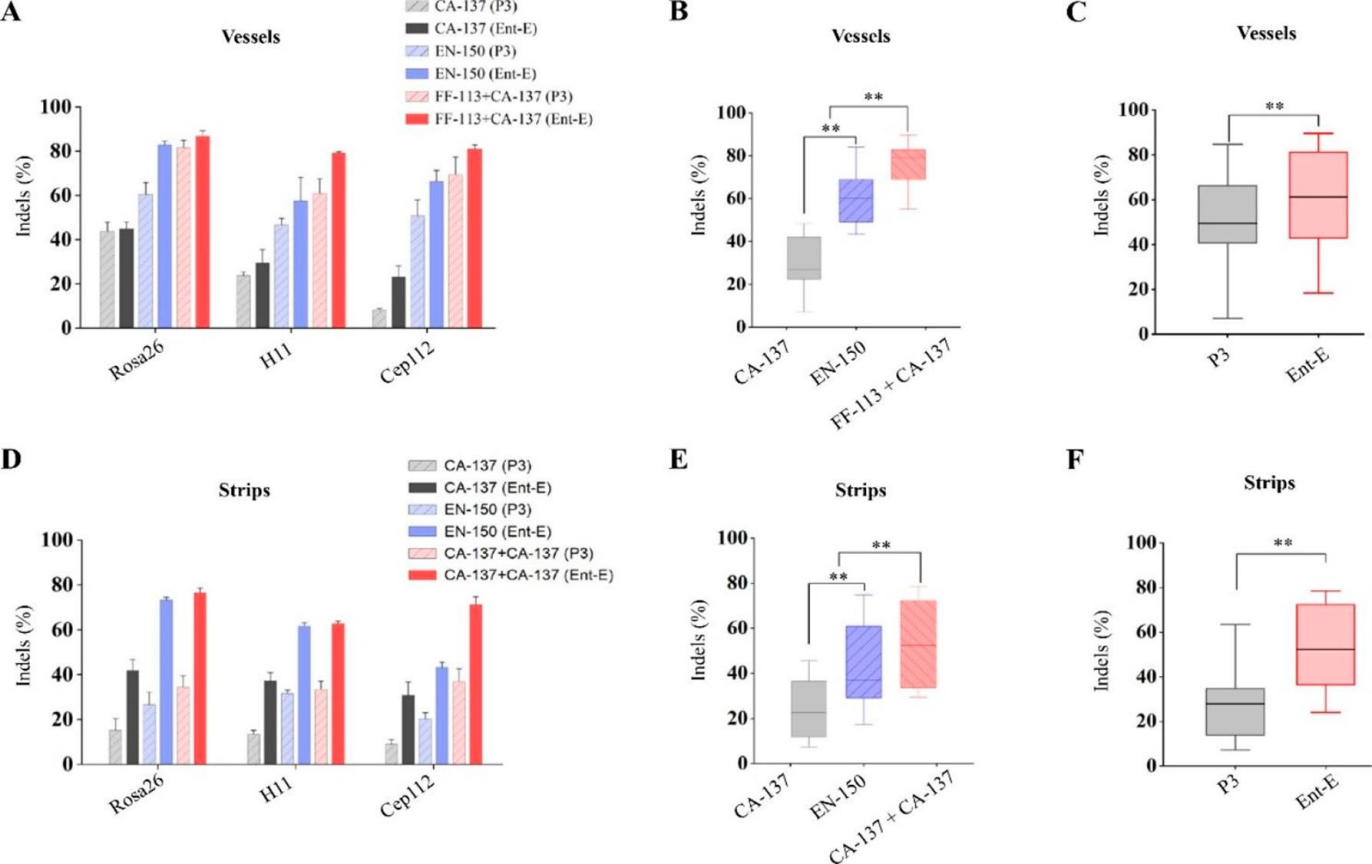
**Indel efficiencies using Entranster-E and P3 buffers with different programs in Nucleocuvette**^**™**^**vessels and strips** (**A**) Indel efficiencies regarding 3 loci in PFFs in vessels. (**B**) Summarized indel efficiencies with different programs in vessels. (**C**) Summarized indel efficiencies using Entranster-E and P3 buffers in vessels. (**D**) Indel efficiencies regarding 3 loci in PFFs in strips. (**E**) Summarized indel efficiencies with different programs in strips. (**F**) Summarized indel efficiencies using Entranster-E and P3 buffers in strips. Ent-E: Entranster-E, **P < 0.01

Ten µg each of CRISPR/Cas9-sgRNA targeting *Rosa26, H11, and Cep112* were delivered using the vessels with P3 or Entranster-E buffer via the FF-113 + CA-137 dual-electroporation program and two single-electroporation programs. The results showed that the FF-113 + CA-137 program combined with buffer Entranster-E resulted in higher than 80% of average indel efficiencies for the 3 genes (Fig. 4A), which is significantly higher (*P* < 0.01) than that of program EN-150 or CA-137 (Fig. 4B). Buffer Entranster-E out-performed buffer P3 for all the 3 genes (Fig. 4C).

In addition, 1 µg each of CRISPR/Cas9-sgRNAs targeting these 3 loci were delivered using the strips with the CA-137 + CA-137 dual-electroporation program and the single-electroporation programs that used for vessels. The results revealed that an average of 70.2% of indel efficiencies for these 3 loci were obtained using the CA-137 + CA-137 program with buffer Entranster-E (Fig. 4D), and the CA-137 + CA-137 dual-electroporation program achieved significantly higher (*P* < 0.01) indel efficiencies than that of the 2 single electroporation programs (Fig. 4E). Electroporation with buffer Entranster-E exhibited significantly higher indel efficiencies for all these 3 loci than that with buffer P3 (Fig. 4F).

## Discussion

As a powerful electroporation system, Lonza 4D-nucleofector™ has been shown to be efficient and reliable in various mammalian cell lines [21, 22]. However, as the Lonza 4D-nucleofector™ manufacturer only provides name codes of built-in programs, thus it is impossible for the user to modify the electroporation conditions such as voltage, pulse width, and number of pulses, unlike other nucleofectors such as BTX [23] and Gene Pulser Xcell [24]. In this study, we firstly detected the transfection efficiencies and survival rates of delivering the 13 kb Cas9-EGFP plasmid and equimolar amount of the 3.5 kb pMAX-GFP plasmid. For the delivery of Cas9-EGFP (13 kb) plasmid, the highest transfection efficiency was obtained via the EN-150 program, and the CA-137 program yielded the lowest transfection efficiency and the highest cell survival rate in both vessels and strips (Figs. 1A and 2A). For the delivery of 3.5 kb plasmid, EH-100 and EN-150 achieved superior transfection efficiencies and comparable survive rates in both vessels and strips (Figs. 1B and 2B). The strips showed overall slightly higher transfection efficiencies and slightly lower cell survival rates than those in vessels for delivering the 13 kb plasmid (Figs. 1A and 2A), whereas the trend was contrary with the delivery of the 3.5 kb plasmid (Figs. 1B and 2B). This suggests that a preliminary experiment for comparison of the transfection efficiency and survival rate in specific cell lines in vessels and strips may be worthy before large-scale electroporation. The cell transfection efficiency usually decreases with the increase of vector size [25, 26]. Indeed, the transfection efficiencies with Cas9-EGFP were notably lower than those for pMAX-GFP plasmid in both vessels (Fig. 1A and B) and strips (Fig. 2A and B) in this study.

Inspired by using repeated electric pulses in BTX nucleofection, we speculated that a combination of single programs might improve the transfection efficiency. To test this hypothesis, we designed 6 custom dual-electroporation programs each consisting of 2 built-in programs executed successively. All the tested dual-electroporation programs yielded transfection efficiencies exceeding 80% for the 13-kb plasmid in either vessels or strips, at the cost of reduced survival rate (Figs. 1C and 2 C). It is worth mentioning that 98.1% of transfection efficiency was achieved when electroporated with FF-113 + CA-137 program in vessels (Fig. 1C). Overall, combinations of FF-113 + CA-137 and CA-137 + CA-137 seemed to have satisfactory performance in delivering the large plasmid (Figs. 1C and 2 C). Using these programs, we found that, for the large size plasmid, there is an optimal range of plasmid amount for transfection of PFFs, taking both transfection efficiency and survival rate into consideration (Figs. 1E and 2D). Therefore, as also mentioned in other studies [27], plasmid dosage is one of the vital parameters for delivering large-size plasmids into hard-to-transfect cells. In addition, we performed CCK-8 assay to assess whether dual electroporation affects the cell proliferation, and found that cell proliferation after electroporation was significantly associated with survival rates but not dual electroporation (Fig. 2, S4).

The Lonza P3 Primary Cell 4D-Nucleofector™ X kit are costly. The provided buffer from the kit is only enough to perform one time electroporation for matched vessels and strips, which are re-usable. Finding an alternative electroporation buffer would significantly lower the cost of electroporation using this nucleofector. We tested 9 alternative candidates, and found that the Entranster-E buffer showed comparative transfection efficiencies and cell survival rates to the P3 buffer when delivering 13 kb plasmid (Fig. 3), suggesting that Entranster-E is suitable for electroporation of large-size plasmid in PFFs. Entranster-E is one type of electroporation buffer available from Engreen Biosystem Ltd (https://www.engreen.co.nz/, China) that is cost-friendly. Based on our purchase record, the cost per reaction using Entranster-E was a fraction of P3 buffer. Furthermore, the results demonstrated a broader range of alternative electroporation buffer choices when delivering a 3.5 kb plasmid (Figure S1A), suggesting that different buffers have distinct transfection effects depending on the size of the plasmid.

Finally, we used two dual-electroporation programs (FF-113 + CA-137 in vessels and CA-137 + CA-137 in strips), and two single-electroporation programs with the highest transfection efficiency (EN-150) and highest survival rate (CA-137) in both vessels and strips, to deliver CRISPR/Cas9-sgRNAs targeting three safe harbor loci to verify the feasibility and advantage of using a dual-electroporation program in a realistic process. The dual-electroporation program yielded significantly higher (*P* < 0.01) indel efficiencies than the single-electroporation programs in all the three targeting loci using vessels and strips (Fig. 4, Figure S2, Figure S3). Genotyping data of single-cell colonies demonstrated that dual-electroporation program achieved overall superior total editing efficiencies as well as biallelic mutant efficiencies than those of single-electroporation programs (Figure S5). Interestingly, the genome-editing efficiencies in examined single-cell colonies were somehow superior to that detected in cell populations (Fig. 4D and E F; Figure S5), suggesting that puromycin selection skewed the single cell cloning outcomes toward nucleofected cells by eliminating non-electroporated cells. Improving the genome editing efficiency has been the major goal regarding CRISPR/Cas9 technology, and a variety of strategies have been employed for this purpose[28–30]. In this study, we achieved a significant improvement of genome editing efficiency by elevating the transfection efficiency. Our study indicates that transfection efficiency is a worth considering factor for genome editing experiments in mammal cells. In addition, the overall indel efficiency generated with buffer Entranster-E was significantly higher than that with the P3 buffer (Fig. 4), though no significant difference in terms of transfection efficiencies and cell survival rates between these two buffers was found (Fig. 3B, C; Figure S1 B, S1 C). Regrettably, we have no detailed information of the composition of buffer P3 or buffer Entranster-E, thus the underlying reason for this discrepancy remains unknown. However, this result suggests that optimizing the composition of the buffer could be an effective approach to enhance the genome editing efficiency. As our data showed that the vessels achieved better transfection efficiencies as well as genome editing efficiencies than those in strips using the customized dual-electroporation programs (Figs. 1, 2 and 4), and in consideration of the capacities of vessels (1 × 10^^6^-10^^7^ cells per reaction) and strips (16 wells, 1 × 10^^4^-10^^6^ cells/well), we recommend using vessels when high transfection efficiencies are required or a large amount of cells are need in one reaction. Otherwise, use of strips may be preferred when a large number of electroporation reactions are needed and relatively lower transfection efficiency is acceptable.

## Conclusions

In summary, our study indicated that customized dual-electroporation programs based on the built-in single-electroporation programs can significantly improve the transfection efficiencies as well as the indel efficiencies via both Lonza 4D-Nucleofector™ vessels and strips in PFFs. Entranster-E can be used as an ideal alternative buffer to Lonza P3 buffer.

## Methods

### Isolation of PFFs

PFFs were isolated from the 30-day-old fetuses of a Large White pig purchased from Fujian Yongcheng Agricultrual & Animal Husbandry Science and Technology Group Co., Ltd (Jiangxi Province, China). Briefly, the sow was euthanized by intravenous injection of potassium chloride solution (100 mg/kg) under anesthesia by an intramuscular injection of 5 mg/kg of Zoletil®50 (Virbac, France) and 1.0 mg/kg of Xylazine (Jilin Huamu Animal Health Products Co., Lt, China). Then the fetuses were harvested and PFFs were isolated from these fetuses using 200 U/ml collagenase type IV (Sigma-Aldrich, USA).

### Preparation of PFF Cell suspension

The 13 kb Cas9-EGFP was a gift from David Feldser (Addgene plasmid, #82,416). The PX459 V2.0 was a gift from Feng Zhang (Addgene plasmid, #62,988). The plasmid DNA was extracted using Endo-free Plasmid Maxi Kit (Qiagen, Germany) according to the manufacturer’s instructions. The plasmid concentration was normalized to 1 µg/µL unless otherwise mentioned. The 3.5 kb pMAX-GFP plasmid (1 µg/µL) was provided in the 4D-Nucleofector™ X kit L (Lonza, Germany). PFFs were cultured in Dulbecco’s modified Eagle’s medium (DMEM, Gibco, USA) supplemented with 12% fetal bovine serum (ExCell Bio, Australia), 100 IU/ml penicillin, and 100 µg/ml streptomycin in a humidified atmosphere containing 5% CO_2_ at 37℃. When cell confluency reached 80–90%, cells were detached with 0.125% Trypsin (Gibco, USA) and counted with a hemocytometer. Cells were pelleted by centrifuging 5 min at 200×g, then resuspended in electroporation buffer (P3 from the P3 Primary Cell 4D-Nucleofertor™ X kit or other buffers stated below). The P3 Primary Cell 4D-Nucleofertor™ X kit L (Lonza, Germany) was used for electroporation in Nucleocuvette™ vessels, and P3 Primary Cell 4D-Nucleofector™ X kit S (Lonza, Germany) was used for electroporation in 16-well Nucleocuvette™ strips. For each electroporation reaction in vessels, 1 × 10^6^ cells were transfected in 100 µL of electroporation buffer containing 10 µg Cas9-EGFP or equimolar amount of pMAX-GFP (2.65 µg) plasmid; for each electroporation reaction in strips, 1 × 10^5^ cells were transfected in 20 µL of electroporation buffer containing 1 µg Cas9-EGFP or equimolar amount of pMAX-GFP (0.265 µg).

### Electroporation program optimization for a large-sized plasmid

The 3.5 kb pMAX-GFP plasmid and the 13 kb Cas9-EGFP plasmid were used to determine transfection efficiency under various electroporation conditions. The seven prestored electroporation programs (CA-137, CM-138, FF-113, EN-150, EO-114, EH-100, and DS-150) recommended for mammalian fibroblasts (https://bioscience.lonza.com/lonza_bs/CH/en/document/21242) were each executed in 100 µL vessels or 20 µL strips with a 4D-Nucleofector™ X unit. As the transfection efficiency for large-sized Cas9-EGFP plasmid was apparently lower, another set of experiments was performed by consecutively executing two of the programs for the same vessel or strip (dual-electroporation). A portion of cell suspension (no plasmid) that was not electroporated served as a control. All experiments were carried out in technical triplicates. After electroporation, cells in vessels were transferred to 60 mm petri dishes containing 5 mL culture medium, and cells in strip wells were transferred to 24-well plates containing 1 mL culture medium. Twenty-four hours post-transfection, the cells were digested and resuspended in the culture medium. Approximately 20% of cell suspension was used for cell counting with a hemocytometer (technical triplicate). The 24 h survival rates were calculated as the percentage of viable cells in electroporated groups compared with the control group. The remaining cells were used for detecting fluorescence by an Accuri C6 flow cytometer (BD, USA), and the transfection efficiency was analyzed with BD Accuri C6 software (Version 1.0.23.1).

### Electroporation buffer and DNA amount optimization

Nine buffers, including PBS (Gibco, USA), DMEM (Gibco, USA), Opti-MEM (Gibco, USA), RIPA-1640 (Gibco, USA), Cytoporation Medium T4 (BTX, USA), K562 (home-made), 1 M (home-made), Ingenio® Electroporation Solution (Mirus, USA) and Entranster-E (Engreen biosystem, China), were tested as alternatives to the Nucleofector™ P3 solution with the FF-113 + CA-137 program for 10 µg Cas9-EGFP plasmid and EN-150 program for 2.65 µg pMAX-EGFP plasmid. Different amounts of Cas9-EGFP plasmid DNA (5, 10, 20, 50 µg for vessels, and 0.25, 0.5, 1, 2, 4 µg for strips) were delivered into the PFFs with the optimal dual-electroporation programs to assess the effect of plasmid concentration on transfection efficiency and cell survival.

### Delivery of CRISPR/Cas9-sgRNAs in PFFs

The CRISPR sgRNAs for porcine Gt (ROSA) 26Sor (*Rosa26*), Hipp11 (*H11*), and centrosomal protein 112 (*Cep112*) were designed using the online software (http://crispor.tefor.net). The annealed DNA oligos of each sgRNA (Table 1) were ligated into the *Bbs*I site of PX459V2.0. The PX459V2.0-sgRNA plasmid (targeting) DNA was extracted by Endo Free Plasmid Maxi kit (Qiagen, Germany). To assess the genome editing efficiency in PFF via the 4D-Nucleofector™ system, 1 × 10^6^ cells were electroporated using vessels with the mixture containing 100 µL of electroporation buffer and 10 µg of targeting DNA, and 2 × 10^5^ cells were electroporated using strips with the mixture containing 20 µL of electroporation buffer and 1 µg of targeting DNA. After electroporation, the cells were transferred into a 6-well plate (regarding vessels) and a 24-well plate (regarding strips), respectively. After 48 h of electroporation, the cells were treated with puromycin (Sigma-Aldrich, USA) at a final concentration of 3 µg/mL for 48 h. Then the culture medium was renewed, and the cells were cultured for another 3 days. Then the cells were collected and digested in 25 µL of lysis buffer (0.45% NP40 and 6 µg/µL of Proteinase K) at 56℃ for 90 min then 95℃ for 15 min. The lysate was used as a template for PCR using PrimeSTAR® Max DNA Polymerase (Takara, China) with primer pairs across the targeting regions of these 3 genes (Table 1). The touchdown PCR conditions were as follows: 95 °C for 3 min; 26 cycles of 95 °C for 30 s, 68 °C (− 0.5 °C/cycle) for 30 s, 72°Cfor 1 min; 14 cycles of 95 °C for 30 s, 55 °C for 30 s, 72 °C for 1 min; 72 °C for 10 min. The PCR products were sequenced on the 3130XL Genetic Analyzer (Applied Biosystem, USA). The TIDE (Tracking of Indels by DEcomposition) web-based software (https://tide.nki.nl/) was used to determine the efficiency of genome editing. The parameters were set to the default values except that the *P* value was set to 0.05 and the indel range size was set to 30 bp.

In addition, we performed another set of transfection experiments targeting the 3 genes and isolated single-cell colonies. In brief, 4 × 10^5^ cells were electroporated using strips containing a mixture of 20 µL Entranster-E buffer and 1 µg targeting DNA. After two days of puromycin selection (described above), the cells were plated into 10-cm petri dishes with various cell densities. After 9–10 days of culture, the single-cell colonies were collected and cultured in 24-well plates, then each cell colony was collected and digested for further PCR detection with primers and conditions as described above.

**Table 1:** sgRNAs and PCR primers used in this study

Name	Sequence (5’-3’)
*Rosa26-*sgRNA	GTGTCGCAATTTCCTGATGA AGG^1^
*H11-*sgRNA	TAGGGGACTAATGAAGTACC AGG^1^
*Cep112*-sgRNA	ACGCTACATGCATATCCTAC AGG^1^
*Rosa26*-PCR-F	AGGGGATTGAGCAGGTGTA
*Rosa26*-PCR-R	CTAAAACAGGTGAGGAGAAAGC
*H11*-PCR-F	TGGCTAATGGCTTTGCTTG
*H11*-PCR-R	TGGTTTTGAGTCCCCTTTCC
*Cep112*-PCR-F	CTGTGCCGTTTGGTTGAG
*Cep112*-PCR-R	CCACTCTCCCCGTTTTCAT

^1^The PAM sequences for sgRNAs are underlined

### Cell proliferation assay

About 1 × 10^5^ cells were electroporated using strips containing a mixture of 20 µL Entranster-E buffer, 1 µg pMAX-GFP or Cas9-EGFP plasmid. After electroporation, the cells were evenly seeded into wells of 96-well plates, and the culture medium was renewed with 100 µL fresh medium and 10 µL CCK-8 solution (Dojindo, Japan) at 7 h, 1, 2, 3, 4, and 5 days (each reaction was performed in technical triplicate). Then the cells were incubated at 37℃ for one hour and absorbance at 450 nm was measured using an automatic microplate reader (TECAN, Infinite M200 PRO, Switzerland).

### Statistical analysis

All data were presented as mean ± SD. The data regarding optimization of electroporation conditions were analyzed by an independent two-tailed student’s t-test. For gene editing experiments, a multi-factor analysis was used, comprised factors including genes, buffers and programs. All data analyses were performed using R v2.14.0 package. *P* < 0.05 was considered statistically significant and *P* < 0.01 was considered statistically extremely significant.

### Electronic supplementary material

Below is the link to the electronic supplementary material.

Supplementary Material 1

## Data Availability

The datasets generated and/or analyzed during the current study available from the corresponding author on reasonable request.

## Abbreviations

PFF: Porcine fetal fibroblasts
SCNT: Somatic cell nuclear transfer
PEFs: Porcine ear fibroblasts
