# Supplementary material for: Efficient delivery of a large-size Cas9-EGFP vector in porcine fetal fibroblasts using a Lonza 4D-Nucleofector system
Source: BMC Biotechnol. 2023 Aug 16;23:29. doi: 10.1186/s12896-023-00799-1 (PMC10428654; doi:10.1186/s12896-023-00799-1)
Supplement: Supplementary file 1 — Supplementary Material 1 [file 12896_2023_799_MOESM1_ESM.doc]

**Supplementary Files**


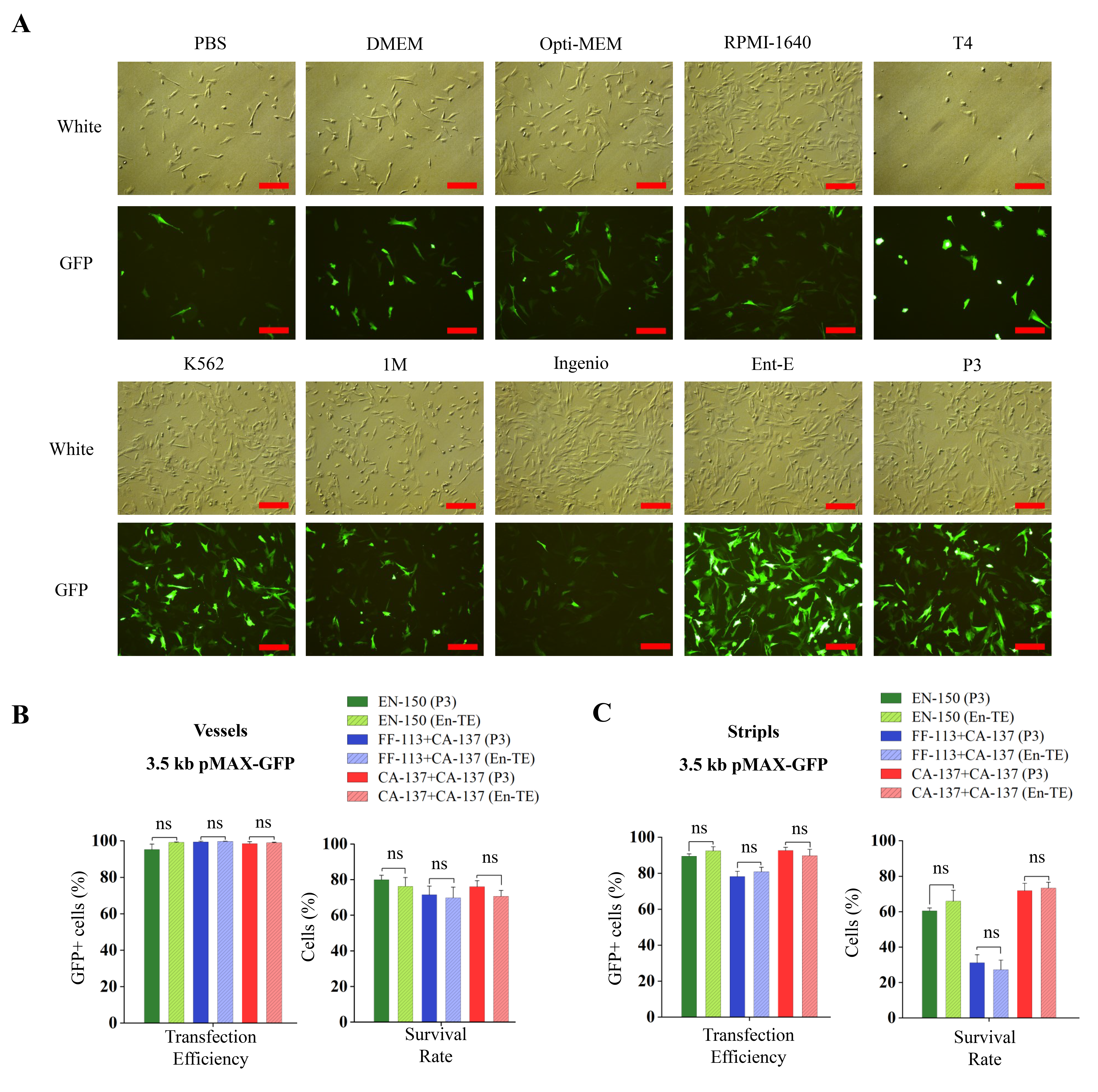
**Figure S1. Screening for an alternative electroporation buffer by delivering the 3.5 kb plasmid.**

(A) Microscopic photographs of electroporation of pMAX-GFP plasmid into PFFs with different buffers. T4: Cytoporation Medium T4; Ingenio: Ingenio® Electroporation Solution; Ent-E: Entranster-E; P3: Lonza 4D-Nucleofector^TM^ X kit P3 buffer. Bar=200 μm. The cells were electroporated with EN-150 program and the photos were captured 24h post-electroporation. (B) Comparison of transfection effects by deliverying the pMAX-GFP plasmid using Entranster-E and P3 buffers in vessels. (C) Comparison of transfection effects by deliverying the pMAX-GFP plasmid using Entranster-E and P3 buffers in strips. ns: *P*≥0.05.


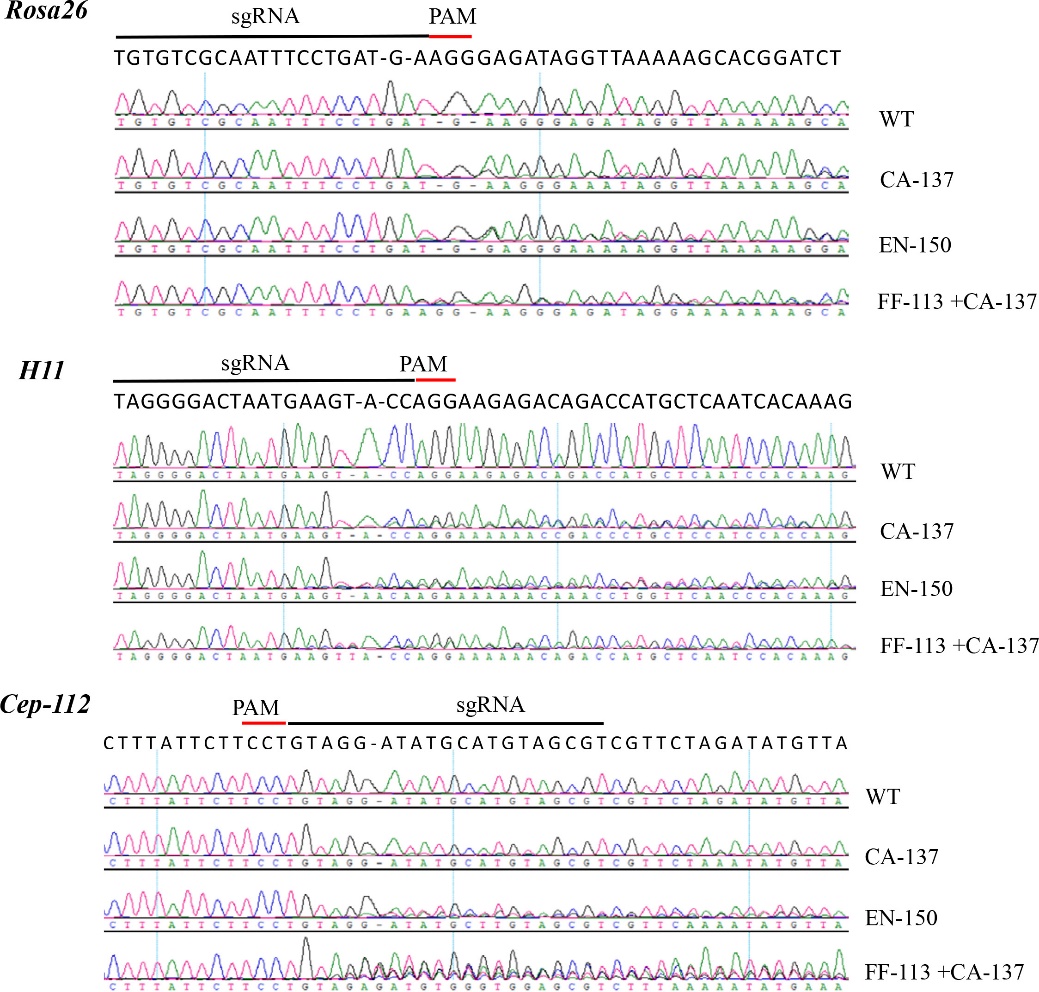


**Figure S2. Sanger sequencing chromatograms for the targeting regions in vessels**

These chromatograms demonstrate indel formation in the three safe harbor loci by using the CA-137, EN-150 and FF-113+CA-137 programs in vessels. The sgRNAs were highlighted with the black lines, and the PAMs were highlighted with the red lines.


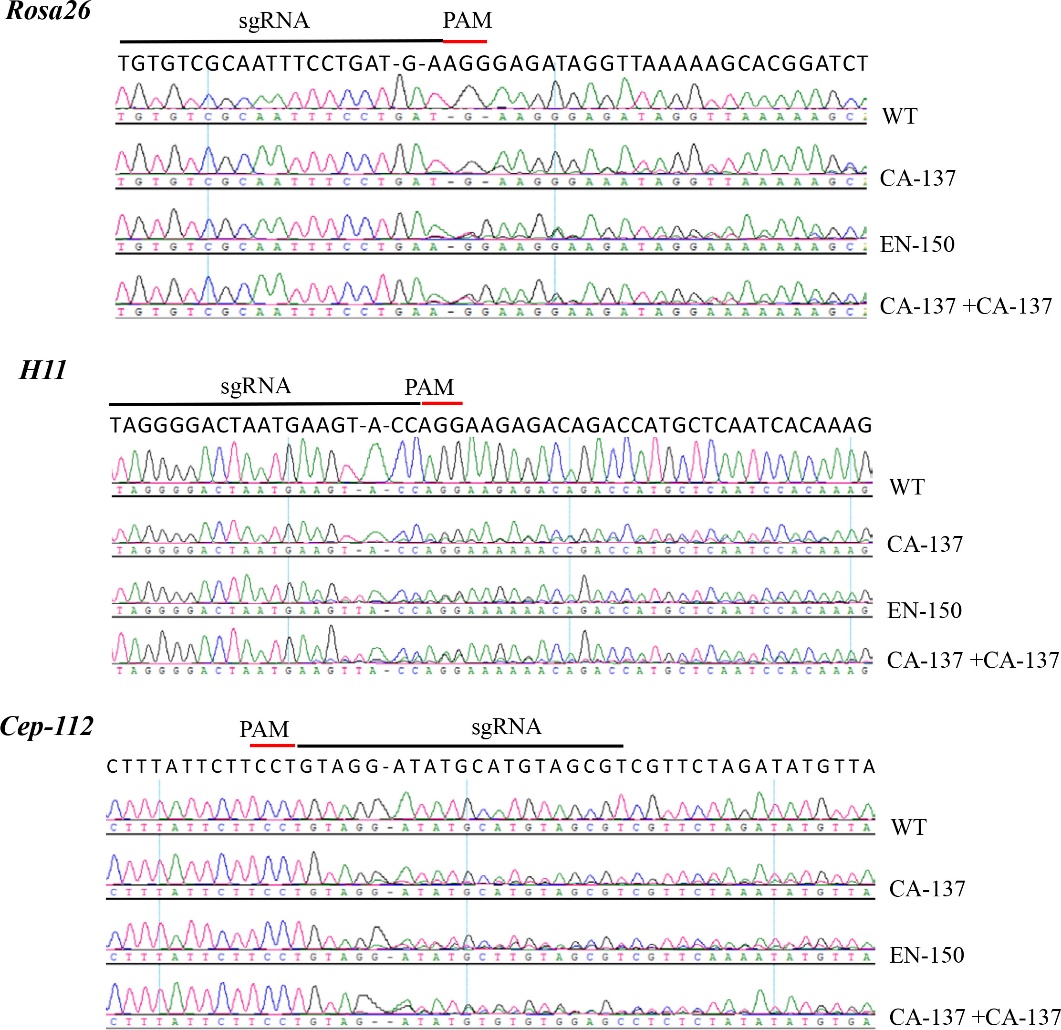


**Figure S3. Sanger sequencing chromatograms for the targeting regions in strips**

These chromatograms demonstrate indel formation in the three safe harbor loci by using the CA-137, EN-150 and CA-137+CA-137 programs in strips. The sgRNAs were highlighted with the black lines, and the PAMs were highlighted with the red lines.


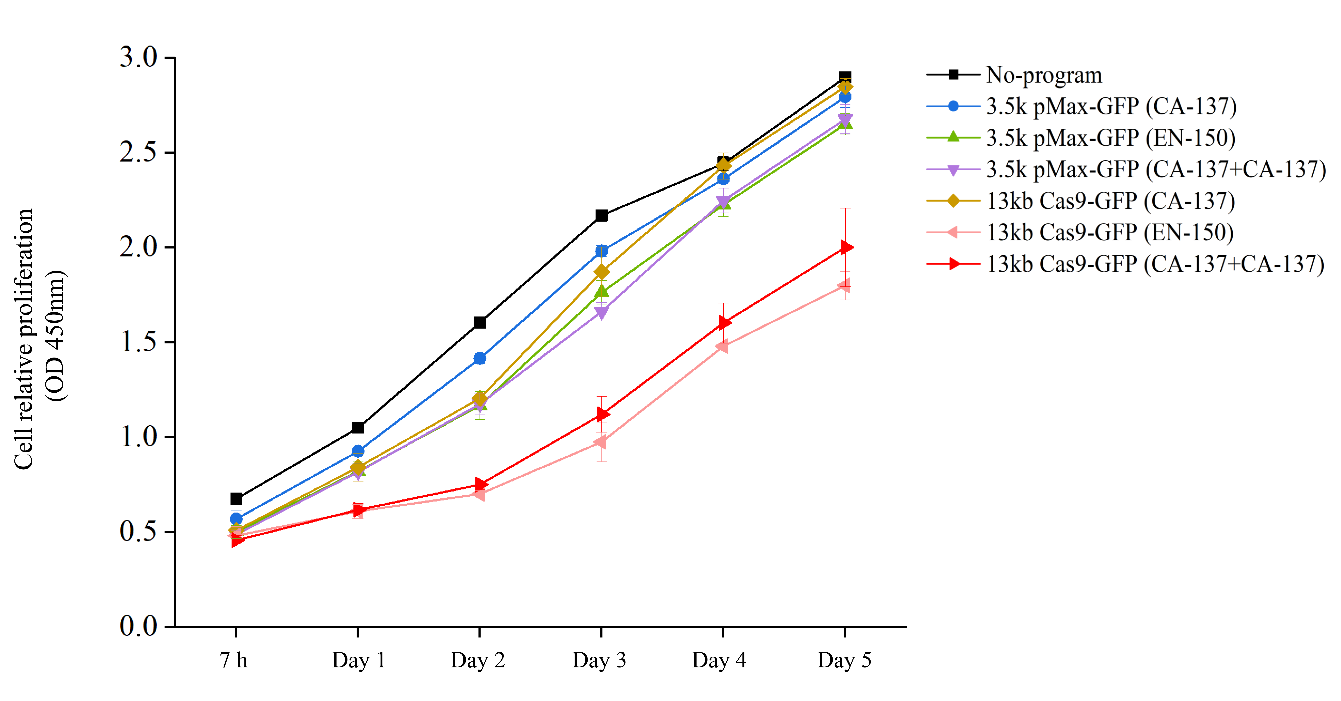


**Figure S4. Proliferation profiles of PFFs electroporated via different programs in strips.**


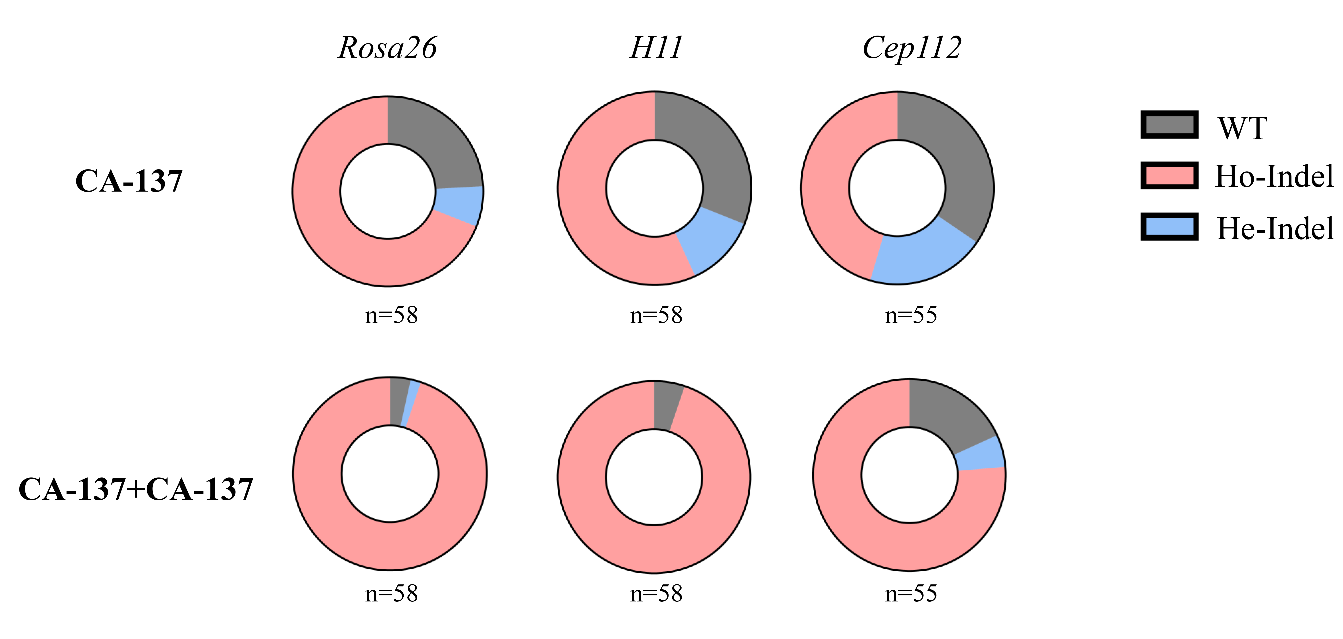


**Figure S5. Indel efficiency of single-cell colonies generated by single and dual-electroporation programs in strips.**

Ho-Indel: Homozygous indel; He-Indel: Heterozygous indel.
